# Magnolol restores the activity of meropenem against NDM-1-producing *Escherichia coli* by inhibiting the activity of metallo-beta-lactamase

**DOI:** 10.1038/s41420-018-0029-6

**Published:** 2018-02-20

**Authors:** Shui Liu, Yonglin Zhou, Xiaodi Niu, Tingting Wang, Jiyun Li, Zhongjie Liu, Jianfeng Wang, Shusheng Tang, Yang Wang, Xuming Deng

**Affiliations:** 1 Key Laboratory of Zoonosis, Ministry of Education, Institute of Zoonosis, College of Veterinary Medicine, Jilin University, Changchun, China; 2 Department of Food Quality and Safety, Jilin University, Changchun, China; 3 College of Veterinary Medicine, China Agricultural University, Beijing, China

## Abstract

The emergence of plasmid-mediated New Delhi metallo-β-lactamase-1 (NDM-1) in carbapenem-resistant Gram-negative pathogens is an increasing clinical threat. Here we report the discovery of an NDM-1 inhibitor, magnolol, through enzyme inhibition screening. We showed that magnolol significantly inhibited NDM enzyme activity (IC_50_ = 6.47 µg/mL), and it restored the activity of meropenem against *Escherichia coli* ZC-YN3, an NDM-1-producing *E. coli* isolate, in in vitro antibacterial activity assays. Magnolol lacked direct antibacterial activity, but compared with meropenem alone, it reduced the MICs of meropenem against *E. coli* ZC-YN3 by 4-fold and killed almost all the bacteria within 3 h. Molecular modeling and a mutational analysis demonstrated that magnolol binds directly to the catalytic pocket (residues 110 to 200) of NDM-1, thereby blocking the binding of the substrate to NDM-1 and leading to its inactivation. Our results demonstrate that the combination of magnolol and meropenem may have the potential to treat infections caused by NDM-1-positive, carbapenem-resistant Gram-negative pathogens.

## Introduction

The emergence and dissemination of multidrug-resistant pathogens, especially Gram-negative bacteria that encode extended-spectrum β-lactamases and are resistant to almost all currently available β-lactam antibiotics, is a worldwide public health problem^1, 2^. Potent carbapenems, such as meropenem and imipenem, were once regarded as the last line of defense against multidrug-resistant Gram-negative bacteria^3^ as they are relatively stable in the presence of most bacterial β-lactamases, including extended-spectrum β-lactamases^4^. However, the increasing use of carbapenems created a vicious cycle that gave rise to carbapenem-resistant Gram-negative pathogens^5, 6^. New Delhi metallo-β-lactamase-1 (NDM-1), classified as an Ambler classB1 metallo-β-lactamase (MBL)^7^ and first identified in *Klebsiella pneumoniae*^8^, is a carbapenemase that was spread globally among multi-drug resistant bacterial pathogens^9^. NDM-1 gives enteric bacteria the ability to inactivate almost all β-lactams, including carbapenems^10^, and this MBL is not inhibited by any existing β-lactamase inhibitors^11–14^. Therefore, the presence of NDM-1 and its closely related variants in *Enterobacteriaceae* is threatening to diminish the effectiveness of carbapenems^15^, forcing the World Health Organization to issue a global warning^16^.

MBLs use one or two zinc ions in their active site to activate a nucleophilic water molecule that cleaves the lactam ring^7^. Although they were first identified half a century ago^17^, many MBLs, such as the Verona integron-encoded MBL (VIM) and imipenemase (IMP) variants, were only present in less pathogenic species, and genes encoding these MBLs were chromosomally located in most isolates. As a result, they have long been neglected in clinical settings^17^. In recent years, the *bla*_NDM-1_ gene was first identified in a transferable plasmid, and soon after, various horizontal gene transfer elements permitted NMD-1 and other resistance genes to spread rapidly and globally^10^. As *bla*_NDM-1_ is one of the major driving forces for the rapid spread of carbapenem resistance that has resulted in a great crisis that threatens the use of β-lactam antibiotics to treat infections caused by NDM-1-producing pathogens, it is considered to be the main target for novel effective inhibitors^18^. Despite the high unmet medical necessity, few effective clinical inhibitors of NDM-1 and other MBLs have been reported so far^19, 20^.

**Fig. 1:**
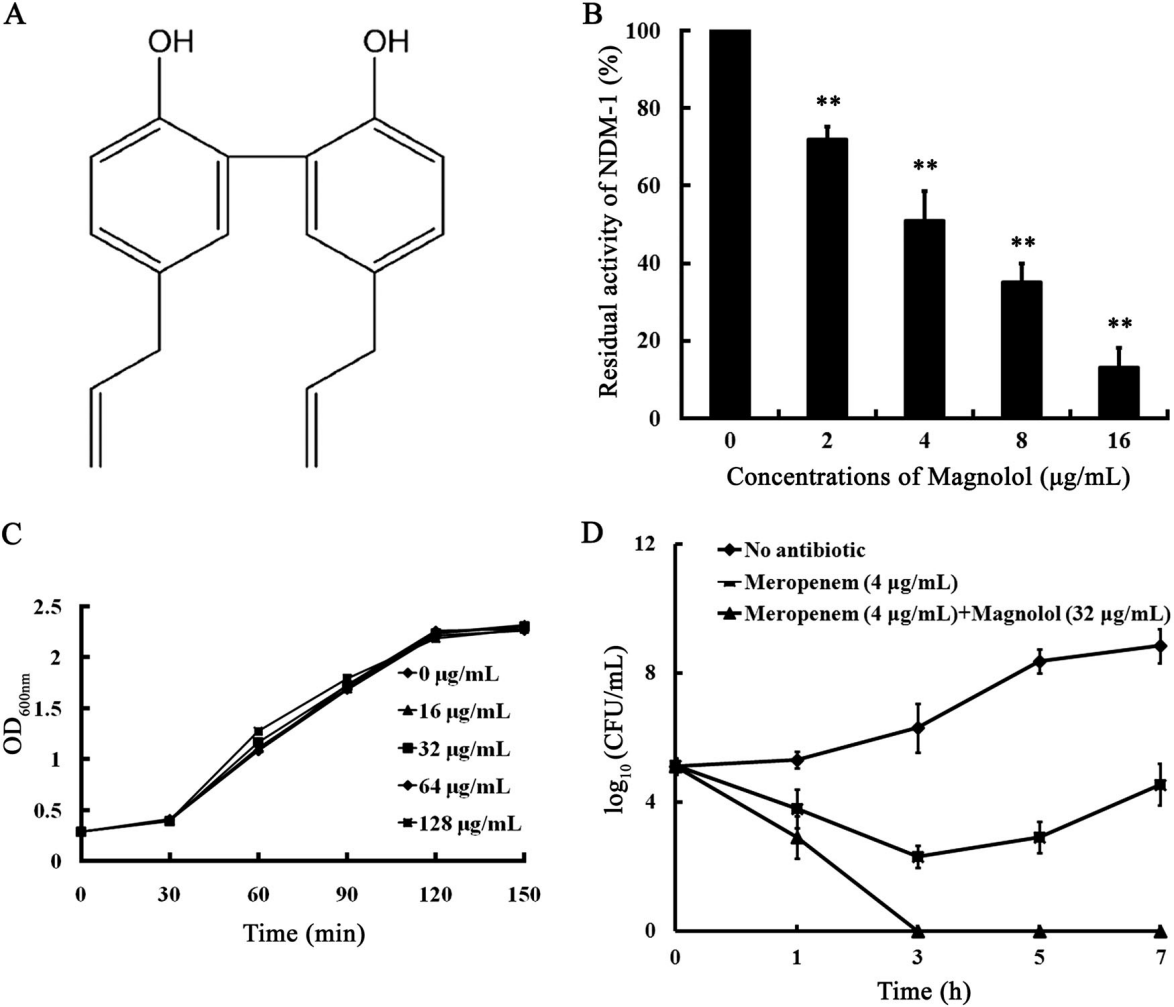
Magnolol-mediated inhibition of *E. coli* ZC-YN3 in vitro. **a** Chemical structure of magnolol. **b** Magnolol inhibits NDM-1 activity. The values are the averages of three independent experiments. ** indicates *P* < 0.01. **c** Growth curves for *E. coli* ZC-YN3 cultured with magnolol. Representative data are one of three independent experiments. **d** Time-kill curves of compounds against *E. coli* ZC-YN3. The values are the averages of three independent experiments

Here we identified a potent inhibitor of the NDM-1 enzyme, magnolol (Fig. 1a), a natural compound isolated from the bark of magnolia (*Magnolia officinalis Rehder and EH Wilson*) trees, using a purified NDM-1 protein and the β-lactamase substrate nitrocefin screening approach. By binding to the active site of NDM-1, magnolol effectively inhibited the biological activity of NDM-1 and successfully rescued the effectiveness of meropenem in vitro against NDM-1-expressing *E. coli*. The combination of magnolol and meropenem may have the potential to treat infections caused by NDM-1-positive, carbapenem-resistant Gram-negative pathogens.

## Results

### Magnolol-mediated inhibition of Escherichia coli ZC-YN3 in vitro

Enzyme inhibition assays using purified recombinant NDM-1 enzymes demonstrated that among 75 natural compounds, only magnolol had a significant impact on MBL enzyme activity in vitro (Fig. 1b). NDM-1 activity (27.92%) was inhibited significantly by adding 2 µg/mL magnolol, and minimal activity (13.24%) was detected in the groups treated with 16 µg/mL magnolol (IC_50_ = 6.47 µg/mL). Moreover, further adding excessive zinc (100 mM ZnCl_2_) to the mixture of magnolol and NDM-1 for 12 h did not restore the NDM-1 activity (data not shown), indicating that the magnolol-mediated inhibition of NDM-1 may not consistent with a metal-depletion mechanism^21^.

Subsequently, magnolol was subjected to antimicrobial testing using *E. coli* isolates in the absence of meropenem. Since Class B1 MBLs generally exhibit significant structural similarity with the ions and disposition of the catalytic residues, inhibitors often work on all MBLs^22^. As shown in Table 1, meropenem plus magnolol reduced the MICs of meropenem against the *E. coli* ZC-YN3 (producing NDM-1), ZC-YN5 (producing NDM-5, two amino acids differ from NDM-1) and ZC-YN7 (producing NDM-9, a single amino acid substitution from NDM-1) by 4-fold compared with meropenem alone. Notably, the fractional inhibitory concentration value of this combination was 0.281, suggesting that these treatments showed synergistic activity. Meanwhile, magnolol alone exhibited no efficient antibacterial effect (MIC > 1,024 μg/mL), and it also had no influence on the growth of NDM-1-producing *E. coli* ZC-YN3 (Fig. 1c).

**Table 1:** MIC (μg/mL) of meropenem against *E. coli* isolates

Strains	Meropenem	Combination
*E. coli* ZC-YN3 (NDM-1)	16	4 (**4**)
*E. coli* ZC-YN5 (NDM-5)	32	8 (**4**)
*E. coli* ZC-YN7 (NDM-9)	64	16 (**4**)

All MICs were determined in triplicate. Magnolol in combination with meropenem was tested at a final concentration of 32 μg/mL. The fold change is indicated in bold

In agreement with the synergy mentioned above, time-kill curves demonstrated that the combination of magnolol plus meropenem exerted killing effects on *E. coli* ZC-YN3, thoroughly killing the bacteria by 3 h after co-incubation (Fig. 1d). Our results indicate that magnolol restored the activity of meropenem against *E. coli* ZC-YN3 in vitro.

### Molecular dynamics (MD) simulation for the NDM-1-magnolol complex

**Fig. 2:**
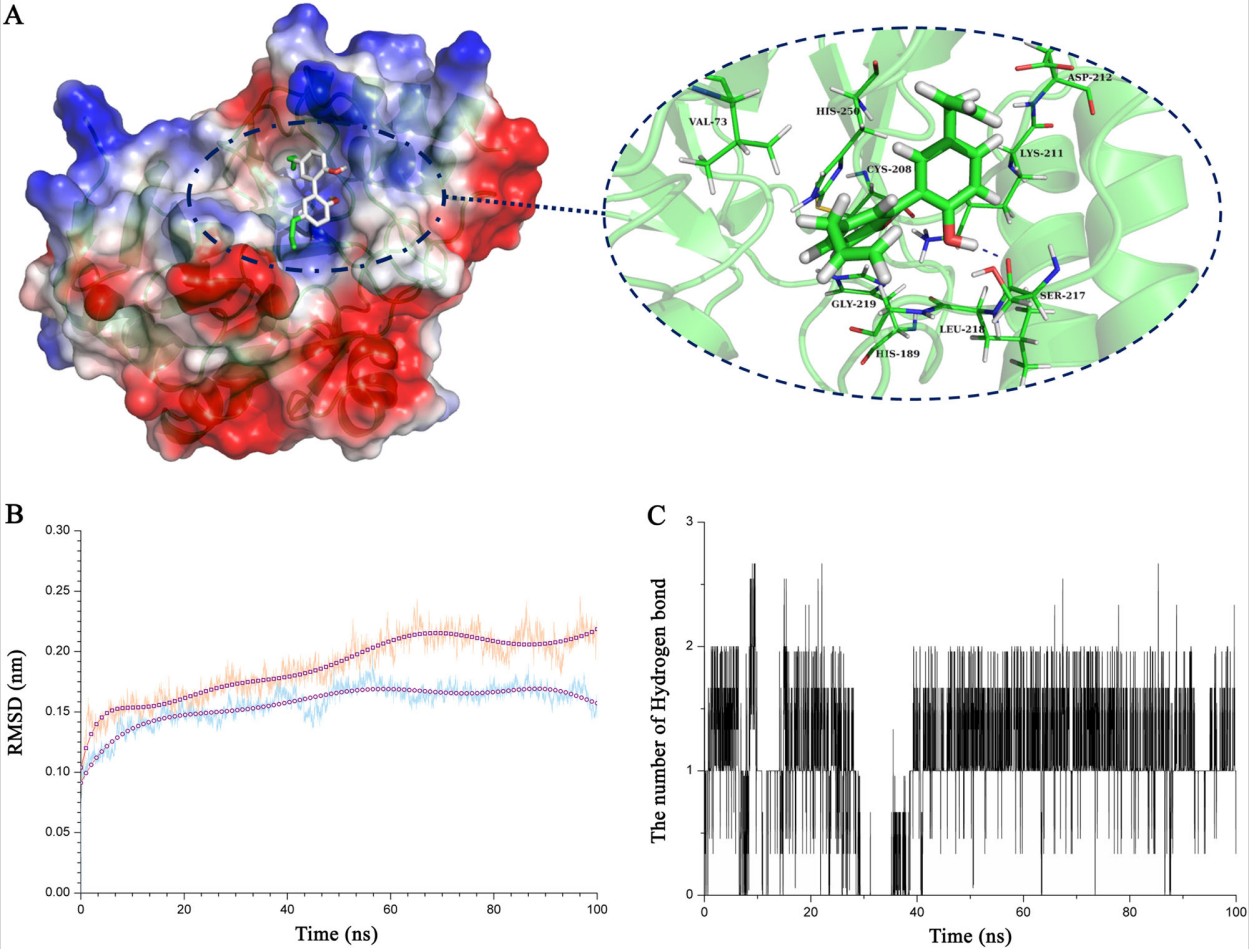
The three-dimensional structural determination of the complex formed by NDM-1 with magnolol by the molecular modeling method. **a** The structure of the NDM-1-magnolol complex. **b** The root-mean-square deviations displayed by the backbone atoms of the protein during MD simulations of the NDM-1-magnolol complex (yellow line) and free protein (blue line) are presented. **c** The number of hydrogen bonds between magnolol and NDM-1 during the simulation

Using a computational biology method, we explored the potential binding mode of magnolol to the active site of NDM-1 (Fig. 2a). It is obvious that magnolol can bind to NDM-1 via hydrogen bonding and hydrophobic interactions. During the time course of the simulation, magnolol localized to the catalytic pocket of NDM-1 (residues 110 to 200). In detail, the binding model of magnolol to NDM-1 revealed that the side chain of magnolol can form one hydrogen bond with Ser217. The complex reached equilibrium at 100 ns based on ananalysis of the root-mean-square deviations of the backbone Cα atoms (Fig. 2b). Moreover, the number of hydrogen bonds was calculated during the simulation. The number of hydrogen bonds fluctuated between one and two during the simulation, further confirming that there is one hydrogen bond between magnolol and NDM-1 (Fig. 2c).

**Fig. 3:**
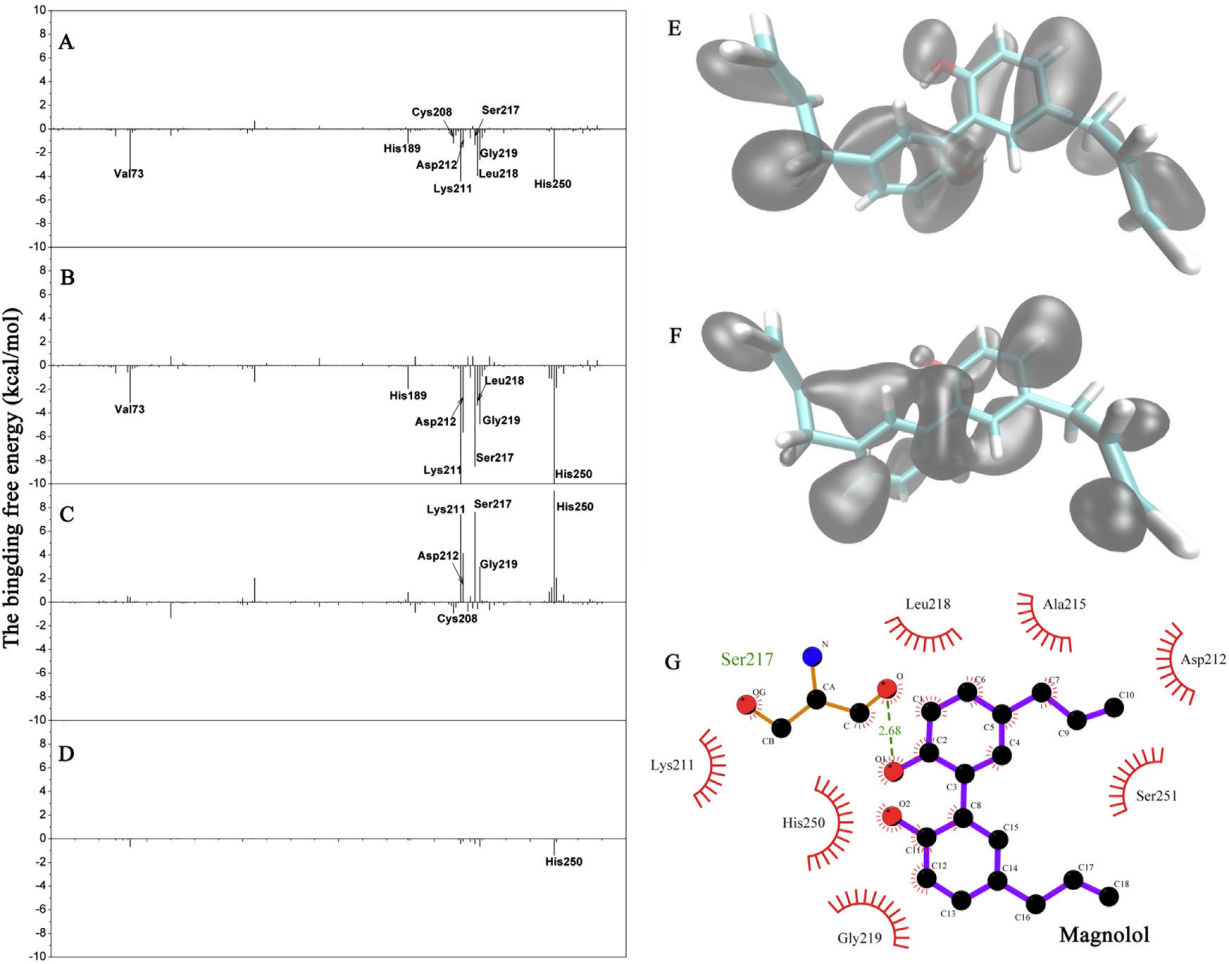
The predicted interaction mechanism between magnolol and NDM-1. Decomposition of the binding energy on a per-residue basis in the WT-NDM-1-magnolol complex. The histogram chart shows the total (**a**), van der Waals (**b**), electrostatic (**c**), and solvation (**d**) contributions for the complexes. The highest occupied molecular orbital (**e**) and lowest unoccupied molecular orbital (**f**) of magnolol. **g** Interactions between magnolol and the residues of the binding sites in NDM-1 are shown using a two-dimensional diagram by Ligplus

To explore the energy contributions from the residues of the binding sites in the NDM-1-magnolol complex, the energy decomposition was calculated for the NDM-1-magnolol complex. Five residues, Val73, Lys211, Leu218, Gly219, and His250, made a strong total binding energy contribution, with a Δ*E*_*total*_ of ≤ −2.0 kcal/mol (Fig. 3a). In addition, residues His189, Cys208, Asp212, and Ser217 also contributed appreciably to the total binding energy, with a Δ*E*_*total*_ of ≤ −1.0 kcal/mol. These results suggest that these five residues (Val73, Lys211, Leu218, Gly219, and His250) are key residues for magnolol binding. As shown in Fig. 3b–d, most of the decomposed energy interaction originated from van der Waals interactions, apparently through hydrophobic interactions, while the electrostatic contribution appeared to have an unfavorable influence on these key residues during complex formation. The magnolol conformation was optimized with the B3LYP/6-311G^*^ set with Gaussian 09 software. As shown in Fig. 3e, f, the highest occupied molecular orbital and the lowest unoccupied molecular orbital of magnolol indicated that the benzene ring is the active binding center of magnolol (Fig. 3g).

**Fig. 4:**
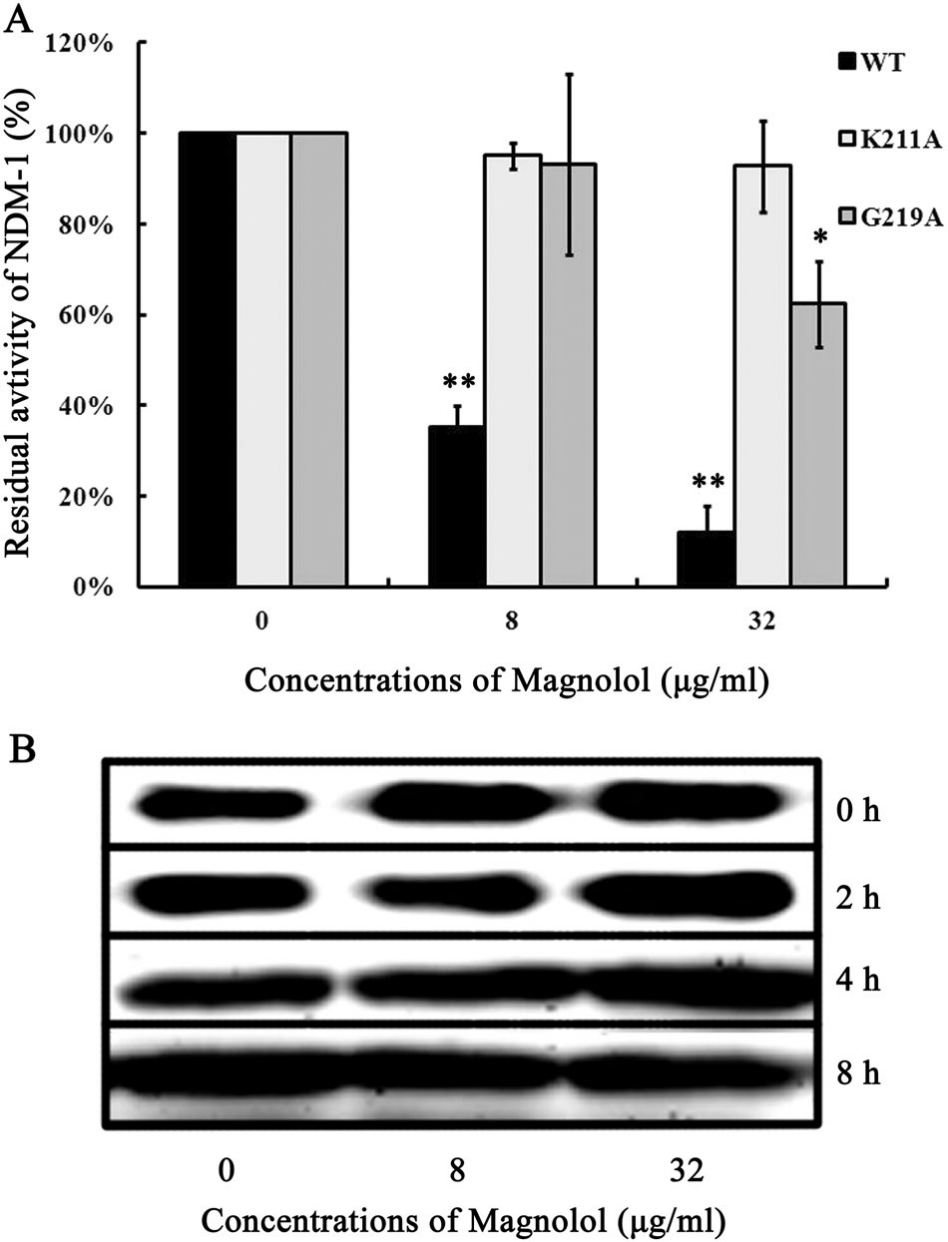
a The effects of WT-NDM-1 and its mutants on NDM-1 activity. The values are the averages of three independent experiments. * indicates 0.01 < *P* < 0.05, and ** indicates *P* < 0.01. **b** The effects of magnolol on the stability of NDM-1

Among these above-mentioned five key residues, since the Val73 is located outside the catalytic pocket and irrelevant for the activity of NDM-1, and the Leu218 and His250 mutant may induce a conformation change in NDM-1 and further affect the binding ability of magnolol to other residues; therefore, we selected the Lys211Ala and Gly219Ala mutants to confirm these theoretical results. The total binding free energy for the NDM-1-magnolol complex and their detailed energy contributions were calculated according to the molecular mechanics Poisson–Boltzmann surface area approach, and they are summarized in Table 2. The binding free energy, Δ*G*_*bind*_, of the interaction between magnolol and wild-type (WT) NDM-1 was greater than that of the Lys211Ala and Gly219Ala mutants, which means that WT-NDM-1 has the strongest ability to bind magnolol. Via fluorescence spectroscopy quenching, we measured Δ*G*_*bind*_ and the number of binding sites between magnolol and the Lys211Ala and Gly219Ala mutants, and these results were highly consistent with those obtained by computational methods (Table 2), further conforming the information generated by the MD simulation of the NDM-1-magnolol complex, namely, that because of the binding of the inhibitor magnolol to the active site region (residues Val73, Lys211, Leu218, His189, Cys208, Asp212, Ser217, Gly219, and His250), the biological activity of NDM-1 was largely inhibited (Fig. 4a).

**Table 2:** The binding free energy (kcal/mol) of the WT-NDM-1-magnolol, Lys211Ala-magnolol, and Gly219Ala-magnolol complexes based on computational methods and the values of the binding constants (*K*_*A*_) based on fluorescence spectroscopy quenching

	WT-NDM-1	K211A	G219A
The binding free energy	−13.6 ± 2.1	−7.9 ± 1.2	−8.6 ± 1.6
*K*_*A*_(1 × 10^4^) L/mol	8.6 ± 1.7	4.8 ± 1.1	5.5 ± 1.3

All tests were determined in triplicate

### Magnolol has no effect on the stability of NDM-1

As shown in Fig. 4b, the stability of NDM-1 was not affected after 0, 2, 4, or 8 h of incubation with different concentrations of magnolol. These data indicate that magnolol has no impact on the stability of NDM-1.

## Discussion

MBLs are the major targets for developing efficient inhibitors against carbapenem-resistant *Enterobacteriaceae*^18^. Through unremitting efforts, various types of MBL inhibitors with different mechanisms have been described. In 2014, King et al. identified a fungal natural product, aspergillomarasmine A (AMA), which is as an effective inhibitor of NDM-1 and VIM-2, through a cell-based screening approach^21^. AMA overcame the resistance mediated by these two MBLs by affecting NDM-1/VIM-2-bound zinc, and it fully restored the antibacterial activity of meropenem. In a mouse model, AMA was shown to be a potential therapeutic agent, but one of the major challenges to its clinical application is the difficulty associated with its chemical synthesis. Fortunately, Liao et al. reported the first total synthesis and stereochemical configuration reassignment of AMA that is amenable to the efficient preparation of AMA^20^. In addition, research by Chiou et al. demonstrated that ebselen, which is an anti-oxidant drug already safely used in human studies, might be a promising inhibitor of NDM-1 by targeting the Cys residue at the active site. However, its anti-oxidant activity and toxicity might limit its potential^23^. Klingler et al. tested 11 approved drugs containing a thiol moiety, and they found four approved drugs (captopril, thiorphan, dimercaprol, and tiopronin) possessed inhibitory activity for NDM-1, VIM-1, and IMP-7. However, the concentrations required to restore their antibacterial activity could not be reached in pathogens^24^. For practical and technical reasons, to date, there are few inhibitors with established potential for clinical application. Thus, the development of MBL inhibitors to restore the activity of β-lactam antibiotics is of great necessity.

Natural compounds have played an important role in the discovery of antibiotics. Magnolol, an abundant natural compound isolated from *M. officinalis*, has been used widely in traditional Chinese medicine^25^. Here we showed that magnolol inhibited the activity of NDM-1, which was confirmed by in vitro experiments. Compared with the above inhibitors, magnolol has the advantages of abundant sources and easy preparation^26, 27^. The high hydrophobicity and low solubility of magnolol may be the major obstacles to its bioavailability and clinical efficacy^28, 29^. However, we revealed that the concentrations required to restore antimicrobial activity could be achieved in bacteria. Moreover, magnolol inhibited NDM-1 activity without impacting NDM-bound zinc, which differs from the metal-depletion mechanisms of AMA. The toxicity associated with cross reactivity with human metallo-enzymes is a major challenge for the development of MBL inhibitors^21^. Known chelators, such as EDTA, have been greatly restricted in clinical use because of these side effects. For instance, the median lethal dose of EDTA was calculated to be 28.5 mg/kg when administered intravenously in mice^30^. Magnolol has little toxicity in vivo because of the differences in its mode of action compared with those of other metal-ion-chelating agents. Animal studies demonstrated that magnolol showed no clinical signs of toxicity in mice and rats^31, 32^. Notably, magnolol has very low toxicity in dogs (no mortality at 1 g/kg when administered intravenously to dogs)^33^. Thus, these data indicate that magnolol may be a potentially safe inhibitor of NDM-1.

In conclusion, our data demonstrate that magnolol inhibited β-lactamase enzymatic activity by binding to the active site of NDM-1, and it restored the activity of meropenem against NDM-1-positive *E. coli* isolates. In vitro, synergistic activity was observed with the combination of magnolol plus meropenem. Taken together, these results identified a potential clinically efficacious dose using *in vitro*, which will contribute to the future development of an effective NDM-1 inhibitor.

## Materials and methods

### Bacterial strains and chemicals

The NDM-1-producing *E. coli* isolates was originated from our previous study^34^. Magnolol (≥98% pure) and meropenem (≥87% pure) were purchased from the National Institutes for Food and Drug Control (Beijing, China). Stock solutions of magnolol were prepared in dimethyl sulfoxide (DMSO, Sigma-Aldrich, St. Louis, MO, USA). Meropenem was dissolved in sterile water.

### Plasmid construction and protein purification

To produce recombinant NDM-1 in *E. coli*, a pET28a-NDM-1 plasmid with the restriction sites BamHI and XhoI was constructed. A *bla*_NDM-1_ gene without the signal peptide was amplified from strain *E. coli* ZC-YN3 with the primers NDM-1-F/NDM-1-R (Table S1). This vector encodes the intact NDM-1 sequence fused to an amino-terminal histidine tag. The Lys211Ala and Gly219Ala mutations of NDM-1 were introduced into pET28a-NDM-1 using the Quick Change Site-directed Mutagenesis Kit (Stratagene, San Diego, CA, USA) with the primers K211A-F/K211A-R and G219A-F/G219A-R, respectively (Table S1). All constructed strains were verified by PCR and sequencing. Protein expression was performed according to Liao et al^20^.

### Enzyme inhibition assays

The assay was conducted according to Liao et al.’s method^20^ with minor modifications. To find NDM-1 inhibitors, we selected 75 natural compounds as screening compounds (Table S2). Assays were read in 96-well plates at an absorbance of 492 nm using a microplate reader (Tecan Austria GmbH, Grödig, Austria) at room temperature. Positive controls were performed in the presence of enzyme and in the absence of inhibitors, whereas negative controls were performed in the absence of enzyme. Residual activity = A−A_0_/A_100_−A_0_ × 100%, where A represents the absorbance of inhibitor groups at 492 nm, and A_0_ and A_100_ represent 0% and 100% activity as determined in the negative controls and positive controls, respectively.

### Antibacterial activity assays in vitro

MICs of magnolol, meropenem, and combinations of magnolol plus meropenem against *E. coli* isolates were determined using the broth microdilution method following the guidelines of the Clinical and Laboratory Standards Institute. The combinations were evaluated by calculating the fractional inhibitory concentration (FIC) index values. To evaluate the effect of magnolol on the growth of the tested strains, a growth curve assay was performed. Specifically, *E. coli* ZC-YN3 was cultured in Luria–Bertani medium at 37 °C with shaking (180 rpm) to an optical density at 600 nm of 0.3 and then aliquoted into five 50-mL conical flasks. Magnolol (or the DMSO control) was added to the five cultures at 0, 16, 32, 64, and 128 μg/mL. The bacteria were cultured at 37 °C with constant shaking, and cell growth was estimated by measuring the OD_600_ every 30 min. In addition, the potential bactericidal effect of magnolol combined with meropenem was evaluated by time-killing assays^35^.

### Molecular modeling

The initial structure of NDM-1 was obtained from the three-dimensional X-ray structure (PDB code: 4EXS). To obtain the starting structure of the magnolol/NDM-1 complex for the MD simulation, a standard docking procedure for a rigid protein and a flexible ligand was performed with AutoDock 4^36, 37^. Subsequently, the MD simulation of the complex was performed. The processes of the computational biology method have been described in detail in previous reports^38, 39^.

### Determination of the binding affinity of magnolol to mutant NDM-1 proteins

The fluorescence-quenching method was used to measure the binding constants (*K*_*A*_) of magnolol with the NDM-1 mutants (Lys211Ala and Gly219Ala). A 280-nm excitation wavelength with a 5-nm bandpass and a 345-nm emission wavelength with a 10-nm bandpass were used for the measurements. Details of the measurements have been described previously^40, 41^.

### NDM-1 stability assays

For NDM-1 stability assays, purified NDM-1 was incubated without magnolol or with 8 and 32 µg/mL magnolol for 0, 2, 4, and 8 h at 37 °C. Western blotting was performed to investigate the stability of NDM-1 treated with magnolol. Anti-histidine-tag antibodies (1:4,000 dilution, Proteintech Group, Inc., Rosemont, IL, USA) and horseradish peroxidase-conjugated goat anti-mouse antibodies (1:2,000 dilution, Proteintech Group, Inc.) were used as the primary and secondary antibodies, respectively.

### Statistical analysis

Data are presented as the mean ± standard deviation from three independent experiments, and they were analyzed using SPSS Statistics for Windows, version 19.0 (IBM Corp. Armonk, NY, USA). Significant differences were determined using an independent Student’s *t*-test as indicated. Differences were considered statistically significant when *P* values were less than 0.05.

## Electronic supplementary material

Table S1 and Table S2
