# Supplementary material for: Magnolol restores the activity of meropenem against NDM-1-producing Escherichia coli by inhibiting the activity of metallo-beta-lactamase
Source: Cell Death Discov. 2018 Feb 20;4:28. doi: 10.1038/s41420-018-0029-6 (PMC5841300; doi:10.1038/s41420-018-0029-6)
Supplement: Supplementary file 1 — Table S1 and Table S2 [file 41420_2018_29_MOESM1_ESM.docx]

**Table S1**. Primers used in this study

| Primer | Sequence 5’ to 3’ |
| --- | --- |
| NDM-1-F | GCGCGGATCCATGCTGAGCGGGTGC |
| NDM-1-R | GCGCCTCGAGTCAGCGCAGCTTGT |
| K211A-F | GCTGCCTGATCGCGGACAGCAAGGC |
| K211A-R | GCCTTGCTGTCCGCGATCAGGCAGC |
| G219A-F | CCAAGTCGCTCGCGAATCTCGGTGATG |
| G219A-R | CATCACCGAGATTCGCGAGCGACTTGG |

The mutated codons are underlined.

**Table S2** List of ineffective compounds (IC50≥ 64 μg/mL)

| Compound name | | |
| --- | --- | --- |
| (-)-Epicatechingallate | Esculentoside A | Maslinic acid |
| (-)-epigallocatechin | Esculin | Matrine |
| Absinthiin | Eugenol | Naringenin |
| Acacetin | Eupatilin | Obacunone |
| Acteoside | Fennel oil | Oroxylin |
| Amentoflavone | Forsythoside A | Perillaldehyde |
| Andrographolide | Geniposide | Phillyrin |
| Anisum stellatum | Ginkgetin | Picfeltarraenin |
| Arctigenin | Ginkgolide A | Potenline |
| Arctiin | Ginsenoside | Quercetin |
| Astilbin | Gypenoside | Rhoifolin |
| Astragaloside A | Hanfangichin B | Rotenone |
| Astragalus Polysacharin | Hesperidin | Rutoside |
| Baicalin | Houttuynin | Schisantherin A |
| Belamcandin | Icariin | Schizandrin A |
| Borneol | Isoalantolactone | Silibinin |
| Brassinolide | Isoforsythiaside | Stevioside |
| Calycosin-7-glucoside | Isofraxidin | Swertiamarin |
| Carvacrol | Isoimperatorin | Taxusin |
| Coixol | Isorhamnetin | Tetramethylpyrazine |
| Coptisine | Jatrorrhizine | Vaccarin |
| Corilagin | L-Epicatechin | Wogonoside |
| Dictamnolactone | Liensinine | Zingerone |
| Dioscin | Luteolin | β-sitosterol |
| Ergosterol | Maleicacid |  |
